# Tea Quality: An Overview of the Analytical Methods and Sensory Analyses Used in the Most Recent Studies

**DOI:** 10.3390/foods13223580

**Published:** 2024-11-09

**Authors:** Juan Moreira, Jyoti Aryal, Luca Guidry, Achyut Adhikari, Yan Chen, Sujinda Sriwattana, Witoon Prinyawiwatkul

**Affiliations:** 1 School of Nutrition and Food Sciences, Louisiana State University Agricultural Center, Baton Rouge, LA 70803, USA; jmoreiracalix1@lsu.edu (J.M.); ajyoti1@lsu.edu (J.A.); acadhikari@agcenter.lsu.edu (A.A.); 2 Department of Food Science and Human Nutrition, Colorado State University, Fort Collins, CO 80523, USA; 3 School of Plant, Environmental, and Soil Sciences, Louisiana State University Agricultural Center, Baton Rouge, LA 70803, USA; lguid53@lsu.edu (L.G.); yachen@agcenter.lsu.edu (Y.C.); 4 Product Development Technology Division, Faculty of Agro-Industry, Chiang Mai University, Chiang Mai 50100, Thailand; sujinda.s@cmu.ac.th

**Keywords:** tea quality, sensory analysis, trained panelists, analytical methods, GC–MS, HPLC, tea grade, chemical composition

## Abstract

Tea, one of the world’s most consumed beverages, has a rich variety of sensory qualities such as appearance, aroma, mouthfeel and flavor. This review paper summarizes the chemical and volatile compositions and sensory qualities of different tea infusions including black, green, oolong, dark, yellow, and white teas based on published data over the past 4 years (between 2021 and 2024), largely focusing on the methodologies. This review highlights the relationships among the different processing methods of tea and their resulting chemical and sensory profiles. Environmental and handling factors during processing, such as fermentation, roasting, and drying are known to play pivotal roles in shaping the unique flavors and aromas of different types of tea, each containing a wide variety of compounds enhancing specific sensory characteristics like umami, astringency, sweetness, and fruity or floral notes, which may correlate with certain groups of chemical compositions. The integration of advanced analytical methods, such as high-performance liquid chromatography (HPLC) and gas chromatography–mass spectrometry (GC–MS), with traditional sensory analysis techniques was found to be essential in the evaluation of the chemical composition and sensory attributes of teas. Additionally, emerging approaches like near-infrared spectroscopy (NIRS) and electronic sensory methods show potential in modern tea evaluation. The complexity of tea sensory characteristics necessitates the development of combined approaches using both analytical methods and human sensory analysis for a comprehensive and better understanding of tea quality.

## 1. Introduction

Tea made from *Camellia sinensis* is one of the most significant and widely consumed beverages in the world [1]. It is estimated that 3.5 billion cups of tea are consumed daily throughout the world [1], with an average annual consumption of 0.43 kg per person in 2022 [2]. Tea consumption has grown quickly during the last two decades [2,3] attributed to mounting evidence of its biological benefits [4,5]. The global tea market is anticipated to show a volume growth of 2.8% in 2025 [2]. The total consumption in the United States has increased from 12.86B in 2021 to 13.9B in 2023 [6].

**Figure 1:**
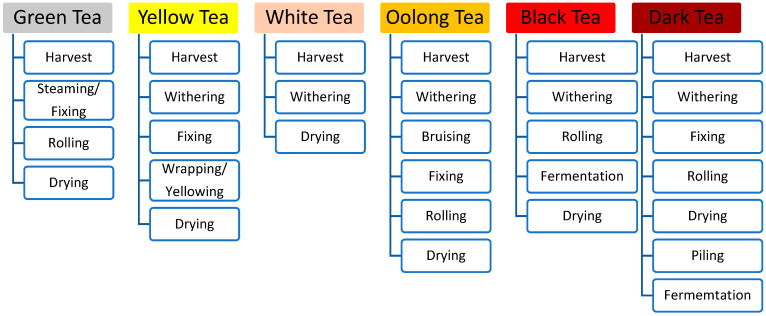
Classification of teas and their common processing steps.

Tea can be broadly divided into six types based on the various processing methods, including dark, black, oolong, yellow, green, and white teas (Figure 1) [4,7]. Their sensory qualities, including the appearance of the dry tea, its infusion color, aroma, and tastes, differ markedly although all teas are produced using the tender shoots of *Camellia sinensis* consisting of one bud and two or three leaves. The different types of tea, and then various name brands and grades within each can have different and unique scents, tastes, and bioactive ingredients since tea contains a large variety of chemicals. The quality of tea is often evaluated by sensory analyses based on human perceptions using sensory descriptors, including morphological traits, tastes, aromas, textural mouthfeel, and colors [8,9]. Moreover, a variety of analytical methods have been developed for tea quality evaluations, including capillary electrophoresis (CE), plasma atomic emission spectrometry, gas chromatography–mass spectrometry (GC–MS), ultraperformance liquid chromatography (UPLC), and near-infrared spectroscopy (NIRS). More recently, the popularity of the electronic eye, tongue, and nose has increased rapidly.

A summary of studies between 2000 and 2020 regarding tea taste, and the analysis and the relationship of tea chemistry and taste, was published by Zhang et al. (2020) [10]. The aim of this review was to provide an overview of various key chemical components associated with tea sensory characteristics, and then, review and discuss the different methods of the identification and quantification of selected compositions with significant health benefits or key characteristics to a type of tea, based on publications between 2021 and 2024. Some novel analytical detection and quantification methods and sensory analyses were suggested. Measuring tea quality indicators is critical for the quality of a final tea product. The findings from this review will help inform tea growers and processors about chemical composition that can be linked to sensory quality and consumer preferences. This could enhance the marketability of teas that meet the consumer and market demands.

## 2. Chemical Composition and Sensory Evaluation of Tea Infusions

One of the most crucial factors to consider when comparing different kinds of tea is aroma. Although the volatile compounds only account for 0.01% of dry weight in tea, they are the important bases for determining the sensory characteristics and economic value of the tea. During processing, volatile substances are altered by enzymatic and thermophysical processes, and account for the majority of the final scent, whereas non-volatile components inherent to the fresh leaves contribute relatively less to aroma [11]. All different types of tea contain various aroma compounds. For instance, the essential aroma components of oolong are linalool and its oxides, benzyl alcohol, 2-phenylethanol, jasmine lactone, and indole. Different brands and grades of oolong tea can be distinguished from one another by the presence of alcohols and esters [12,13]. A study performed with Longjing, a Chinese green tea, identified up to 47 aroma compounds using the stir bar sorptive extraction, gas chromatography-olfactometry (SBSE/GC-O), and odor activity value (OAV). The presence of compounds such as 2,4-heptadienal, 3-methylbutanal, dimethyl sulfide, and geraniol considerably enhanced the intensity of characteristic aromas [14]. Moreover, compounds in Tieguanyin oolong teas such as benzyl benzoate and heptanal, epicatechin and gallate, have been found to be reliable indicators for determining the tea grades, with lower grades having lower intensities of these compounds [15].

HPLC and GC–MS have been widely used to determine the composition and quantity of volatile and non-volatile chemical compounds associated with the taste and aroma of tea. These techniques are valuable tools often used in conjunction with sensory analyses to describe the intensity of sensory attributes in tea infusions based on their composition. Chemical composition and sensory analyses of the six most consumed teas are discussed, beginning with black, dark, green, and oolong teas, followed by yellow and white teas. A summary table is provided at the end of this section (Table 1).

### 2.1. Black Teas

Black tea is the most consumed tea in the world, accounting for ~75% of total consumption. It is known for its bold flavor, rich aroma, high caffeine content, and potential health benefits [16]. Keemun black tea (KBT) is a traditional Chinese black tea, well known for its floral aroma. The aroma compounds in KBT are affected by several factors during processing, including withering, rolling, fermentation, and drying. Su et al. (2022) [17] evaluated volatile compounds of different grades of KBT using GC–MS. Additionally, the five grades of finished KBT, superfine (highest grade), special grade, first grade, secondary grade, and third grade, were evaluated by trained panelists using Quantitative Descriptive Analysis (QDA). Panelists consistently detected a sweet aroma in all five grades, and the intensity of this aroma increased as the grade lowered (larger particles). Contrarily, the higher grades (finer particles) presented higher intensities of “Keemun aroma”, a distinctive honey-like floral and fruity scent specifically found in KBT. Sensory evaluation results were in accordance with volatile compound determinations, where higher grades of KBT contained larger amounts of geraniol, linalool, and methyl salicylate, all of which were considered key attributes to the floral Keemun aroma [17]. Fermentation time, a key factor in flavor and aroma development, has been a driving factor in consumer preference of black tea. A 3 h fermentation duration was identified as the best treatment compared with 2, 4, or 5 h, and it resulted in the highest retention of catechins in a study with Yunnan Congou black tea [18]. Compared to KBT made from leaves plucked in the spring, KBT processed from leaves harvested in summer and fall often yield lower grades with increased bitterness and reduced aroma quality. However, one study indicated that the flavor of KBT was found to be improved by including *Cordyceps militaris*, a fungal with medicinal value, to the solid-state fermentation process, which reduced the total polyphenol, total flavonoids, and total free amino acids of the final KBT product [19]. Additionally, the drying methods affected the sensory and chemical quality and metabolic profile of black teas. As an instance, Congou black tea dried with hot air received higher sensory scores and better chemical qualities (i.e., infusion color and a ratio of polyphenol to amino acids) than hot roller drying, with hot roller drying generating fruity flavors and hot air drying producing sweet and flowery flavors [20]. The effects of drying temperatures during the final processing step on the taste, color and biological capacities of black tea were investigated [21]. They reported that a drying temperature at 100–110 °C yielded the best taste characteristics with higher sweetness and umami scores as well as color appearance, which might be attributed to Amadori products formed in the initial stage of the Maillard reaction. The findings from this study [21] are useful for producing black tea with more ideally flavored, higher antioxidative and anti-hypoglycemic capacity by regulating drying temperatures.

Huang et al. (2022) [22] examined the effect of withering on KBT using sensory evaluation, electric tongue, and HPLC. The study used three types of withering procedures such as natural withering (8–19 °C, 24 h), sun withering (15–17 °C, 2.5 h), and warm-air withering (18–22 °C, 7 h). Although all samples were perceived to be sweet by a sensory trained panel, sun-withered KBT was perceived as more bitter and astringent. Interestingly, the electric tongue analysis determined that sun-withered samples were in fact sweeter and more astringent than the rest due to the following reasons: (1) the sun-withered KBT had a higher glucose concentration due to the increased exposure to the sun which could have increased photosynthesis and sugar breakdown; (2) this sample had a higher concentration of sweet amino acids (proline, cysteine, alanine, serine, and glycine) and lower amounts of theanine which was associated with green tea flavors; and (3) catechins may be the main reason for the highly astringent taste of the sun-withered KBT sample [22]. Moreover, longer withering times, 10 h, but not longer, can also lead to higher soluble sugar and amino acid contents [23]. The results from the trained sensory panel vs. the electric tongue technique evidenced some limitations of the latter technique as a possible replacement for sensory evaluation. This is mainly due to the inability of the electric tongue to account for certain physiological taste interactions perceived by the trained sensory panel such as suppression, for example, bitterness suppressed by sweetness; or synergy among different sensory attributes, such as a combination of bitter compounds and catechins, or monosaccharides and sweet amino acids, that would result in greater perceived tastes in bitterness or sweetness, respectively.

In another study by Huang et al. (2021) [24], metabolites of KBT during storage time were evaluated using HPLC and liquid chromatography tandem mass spectrometry (LC-TMS). In addition, sensory quality was evaluated by a trained sensory panel using the equivalent quantification method. The different tea samples in this study were stored between 1 and 20 years. Samples with a 10-year storage time had a significant reduction in the content of epicatechin and epigallocatechin gallate (EGCG). However, caffeine and theobromine levels were not affected by the storage time. Quinic acid derivatives were important taste contributors for stored KBT. Additionally, the trained panel reported that sweetness increased with years of storage, while the opposite occurred with astringency, bitterness, and umami [24]. Future studies are needed to better understand why fatty acids consistently accumulate in KBT as the storage period extends. Wen et al. (2022) [25] also studied flavor characteristics of KBT, but focused on finding the compound that had the highest impact on the astringency of these teas. Using combinations of mass spectrometry, turbidity analysis, and sensory evaluation, p-coumaroylquinic acids were found to be the key compounds imparting astringency [25]. The use of turbidity analysis in combination with sensory analysis and mass spectrometry may prove to be an effective technique for determining some compounds responsible for specific flavors in tea samples. However, the process still needs to be optimized to solve the current limitations due to complex and diverse compounds in tea infusions.

Black tea sensory attributes are not only affected by its grade or processing method, but also by the country or region. Depending on the origin, certain aromas and flavors are expected by consumers. Wang et al. (2022) [26] evaluated 112 black tea samples from seven countries: China (81), Sri Lanka (22), India (5), Malaysia (1), Kenya (1), Mauritius (1), and the United Kingdom (1) using headspace solid-phase microextraction and gas chromatography–mass spectrometry (HS-SPME/GC–MS). Linalool, pentanoic acid, and hexanoic acid in Indian black teas, phenylethyl alcohol in Chinese black teas, and 1-methyl-naphthalene, *β*-ionone, and methyl salicylate in Sri Lankan black teas are discriminating volatile compounds among countries of origin. Additionally, a team of certified panelists evaluated the samples, and found that sweet and floral aromas predominated most of the black tea samples, which was consistent with GC–MS analysis. Particularly, samples from China and India presented higher intensities for the sweet and floral aromas which may be explained by their higher geraniol, phenylethyl, and linalool contents. The high contents of methyl salicylate contributed to the peppermint aroma of tea samples from Sri Lanka [26]. Among Chinese samples, linalool and benzeneacetaldehyde in Yingde (Guangdong) black tea, methyl salicylate in Taiwanese samples, and benzeneacetic acid in Anhui KBT could be used as biomarkers to distinguish them from other Chinese samples. Similarly, Chen et al. (2022) [27] evaluated the aroma quality of 44 different black teas from the Yunnan region in China, better known as Dianhong black tea (DBT), using the rapid gas chromatography-electronic nose (GC-E-Nose). Alcohols and aldehydes in these samples included 2-methylfuran, 2-methylbutanal, linalool, and 1-hexanol, which generate chocolate, nutty, floral, and fruity aromas, respectively. Additionally, when evaluated by certified tea assessors, 14 out of 44 samples were considered as high quality. Floral, chocolate, and nutty aromas had greater effects on sensory grading and were induced mainly by linalool and 2-methylfuran [27]. This study demonstrated that the GC-E-Nose technology could be supplementary to sensory evaluation, providing a new technique for black tea quality evaluation [27].

There are several famous black teas, including Keemun, Assam, Darjeeling and Ceylon black teas, all of which are produced in lower latitude. Only a few studies evaluated aromatic characteristics of high-latitude black teas. Wang et al. (2021) [28] utilized GC–MS and gas chromatography–olfactometry (GC–O) analyses to evaluate the aroma compounds of six teas from different latitudes to determine suitable cultivars for high latitudes. Although many compounds were found similar in these samples, 12 compounds, (E)-2-octenal, cis-3-hexenyl hexanoate, (E)-2-hexenyl hexanoate, linalool, d-cadinene, octanal, (E,E)-2,4-heptadienal, epoxy linalool, methyl salicylate, 2,6-bis(1,1-dimethylethyl)-2,5-Cyclohexadiene-1,4-dione, ethyl 2-(5-methyl-5-vinyltetrahydrofuran-2-yl)propan-2-yl carbonate, and hexadecane were identified as ideal markers for differentiating black teas from different latitudes. The high levels of methyl salicylate and geraniol in two cultivars, Jinxuan and Longjing Changye, contributed to the highest sensory scores due to their pleasant floral aroma [28]. These two cultivars were ideal for the high-latitude production of high-quality black tea. Jin et al. (2021) [29] also evaluated black tea quality but focused on how to use intellectual devices to automate the fermentation process at an industrial scale. Employing micro-near-infrared spectroscopy (micro-NIRS) and a lab-made computer vision system (CVS), a principal component analysis (PCA)–support vector machine (SVM) model was developed to predict the changes in indicator compounds (i.e., theaflavin) during fermentation at 89.19% accuracy compared to the conventional use of trained sensory panelists and HPLC [29]. Li et al. (2021) [30] and Wang et al. (2021) [31] also used the micro-NIRS method coupled with sensory evaluation and HPLC to analyze the quality grades of KBT, and reported performance accuracies as high as 94.29% with the PCA–SVM model [30]. Such models were able to predict bitterness and astringency scores, and the caffeine content [31]. However, findings in these studies are still at a lab-scale and factors, such as changes in season and variety, must be evaluated to make quality determinations applicable in different situations [29,30,31].

### 2.2. Dark Teas

Dark teas are post-fermented teas processed by steaming, piling, pressing, fermentation, and drying steps. There are different types of dark teas. For example, Fu brick tea (FBT) has special sensory characteristics of fungal aroma formed from a microbial fermentation process [32]. Li et al. (2021) [33] evaluated the dynamic changes in metabolites and taste profiles of FBT at different stages of its fermentation. Using an ultrahigh performance liquid chromatography system coupled to a quadrupole-time of flight mass spectrometer (UPLC-Q-TOF/MS), catechins, flavonoids and flavone glycosides, phenolic acids, and terpenoids were identified as major metabolites. The tea samples were taste-tested every three days after fermentation for up to 22 days by a trained sensory panel to evaluate the intensities of bitterness, astringency, sourness, and mellowness. The panel reported that sweetness was found to remain stable during post-fermentation. As time went by, from 6 days of fermentation onwards, the intensity of astringency, bitterness, and sourness reduced, meanwhile, mellowness intensity gradually increased after 9 days of fermentation onwards [33]. Several catechins and phenolic acids such as flavonoid glycosides were found to be positively related to the astringent, bitter, and sour taste attributes of FBT [33]. These substances in the fresh tea leaves are reformed during the fermentation process of dark tea via the action of microorganisms. Zhao et al. (2024) [32] utilized GC-Q-TOF-MS, GC-O, and the aroma extraction dilution analysis (AEDA) to identify and quantify the key odorants responsible for the fungal aroma in FBT. The 15 key odorants identified were (*E,E*)-2,4-heptadienal, (*E,E*)-2,4-nonadienal, (*E*)-2-nonenal, (*E,Z*)-2,6-nonadienal, (*E*)-2-octenal, (*E*)-*β*-ionone, 4-ketoisophorone, dihydroactinidiolide, (E)-*β*-damascenone, 1-octen-3-ol, linalool, geraniol, heptanal, hexanal, and phenylacetaldehyde.

*Aspergillus cristatum* (*Eurotium cristatum*) was the dominant fungus during the manufacturing process of FBT. Fermentation proves to be indispensable in the production of dark teas due to the critical involvement of fungal genera, including *Aspergillus*, *Candida*, *unclassified-o-Hypocreales*, *unclassified-o-Saccharomycetales*, and *Wallemia*, and the bacterial genus, *Klebsiella*, serving as core functional microorganisms that contribute to the metabolic variations during the fermentation process [33]. In another study on post-fermentation methods for making Liupao tea, a famous dark tea in the Guangxi region of China, traditional piling with manual turning during fermentation was compared with automated tank fermentation technique with the purpose of reducing labor cost and improving fermentation uniformity. The sensory and chemical characteristics of Liupao tea changed significantly during fermentation. The contents of monomeric catechins, polyphenols, flavonoids, free amino acids, water extract, and thearubigins decreased significantly, those of theaflavins remained stable, and those of soluble sugar and theabrownins increased significantly. The content change rate of polyphenols, flavonoids, and theabrownin in traditional fermentation was approximately twice that in tank fermentation, which led to lowered astringency, bitterness, and sourness [34].

In the dark tea market, An tea (AT) after long-term storage has greater commercial value. Shen et al. (2022) [35] studied the changes in AT quality based on metabolomics and sensory evaluation during storage from 2 to 35 years. The taste of the aging tea samples started to change gradually after 7 years of storage. However, the astringency perception did not disappear after 25 to 35 years of storage. The loss of astringency was likely due to the oxidation of flavonoids during long-term storage. Using UPLC-Orbitrap-MS analysis, 12 compounds, quercetin, quercetin 3-O-glucuronide, glycine, aspartic acid, alanine, serine, arginine, threonine, tyrosine, theanine, γ-aminobutyric acid (GABA), and isoleucine, were found to gradually reduce, while quercetin 3-O-rutinoside increased gradually with increasing storage time. These compounds can be used as characteristic markers to help identify the actual age of AT, which is of great interest to consumers and the dark tea market because their contents can be used to determine if the product is ready for the market [35].

### 2.3. Green Teas

Green tea is widely consumed in Asian countries and accounts for about 30% of the global tea consumption. Green tea undergoes fewer processing steps compared to other types of tea, which can mostly preserve the original compounds of fresh tea leaves [36]. Different processing methods, particularly the fixing step, either roasted, baked, sun-dried, or steamed, result in large differences in the flavor of the final product. Steamed green tea has a long history and unique aroma, but little is known about its key aroma components. Qin et al. (2024) [37] identified 173 volatiles in steamed green tea using solvent-assisted flavor evaporation and SPME plus two chromatographic columns of different polarities. Of these, dimethyl sulfide, (*E*)-*β*-ionone, *cis*-jasmone, linalool, nonanal, heptanal, isovaleraldehyde, and (*Z*)-3-hexenol were the key aroma active compounds of steamed green tea. As the degree of withering increased, the content of these compounds increased first and then decreased, except for heptanal and *cis*-jasmone [37]. A study by Zhu et al. (2021) [38] showed that flavonoids, particularly catechins and flavonol glycosides, decreased significantly as roasting temperatures increased. This leads to the roasty and burnt odor of the teas according to the evaluation of trained sensory panelists.

Zhang et al. (2022) [7] evaluated both Chinese black and green teas to attempt to explain the main factors driving a sour taste. Organic acids were thought to be the main contributor to the sour taste; however, they discovered that 11 organic acids had an overall taste activity value (TAV) (a ratio of the substances concentration to the taste threshold) of >1, with succinic acid (sour with a light umami) and citric acid (sour with a gentle astringent) in black tea and succinic acid and lactic acid (sour with a gentle astringent) in green tea being the top contributor. Although the concentrations and TAV values of these acids are comparable in both teas, the sour taste of black teas was more intense and almost non-detectable in the green tea samples. Therefore, sourness may be affected by many factors such as infusion pH and the interactions between different sensory attributes. A clear example of this is the high intensity of bitterness and astringency in green teas, which may suppress their sourness. Conversely, a moderate intensity of bitterness may enhance sourness in black teas.

The aroma profiles of green teas produced from summer tea leaves were evaluated by Guo et al. (2021) [39] to investigate if fresh leaves plucked during summer were suitable for making green tea. They found that L-theanine was an important precursor for the formation of green tea aroma. The significant reduction of L-theanine content after the roasting process indicated that there might be a thermal reaction from which nitrogen-containing heterocyclic compounds formed and contributed to the roasted or nutty aroma in green tea. This was confirmed, by the trained panelists who evaluated the infusions prepared from the roasting process, to have strong notes of fragrance and nutty odors [39]. Han et al. (2022) [40] evaluated six different grades of Huangshan Maofeng green teas to determine which taste-active compounds contributed significantly to bitterness, astringency and sweet aftertaste. Through UPLC-MS based metabolomics, six marker compounds responsible for the grade differentiation were identified as procyanidins, flavonoid glycosides, monogalloyl glucose, digalloyl glucose, trigalloyl glucose, and galloyl-hexahydroxydiphenoyl-glucose [40]. To reduce the “green” flavor in green tea, which is less preferred by Western consumers, Rigling et al. (2021) [41] used edible *Basidiomycetes* and green tea leaves for fermentation. After fermentation, (E, E)-2,4-decadienal, geraniol, and (E)-methyl jasmonate, which are responsible for floral and green aromas, decreased significantly. This was further confirmed by the sensory panel who found that the intensity of green and soapy aromas was decreased or even undetectable after fermentation [41]. The biosynthesis of a nutty and cocoa-like odor by 2-ethyl-3,5-dimethylpyrazine in *Basidiomycetes* after 16 h of fermentation was reported for the first time, which creates an important research opportunity for using *Basidiomycetes* and other microbials to induce chocolate-like and nutty flavor as well as cinnamon, fruity, herbal, and honey odors in green teas, and thus increase its appeal to a broader range of consumers [41]. To further investigate how volatile compounds and the quality of Meixiang green tea change during aging (stored for 1–16 years and 20 years), Liu et al. (2023) [42] studied changes in the microbial community composition of this Meixiang green tea during storage by 16S rDNA analyses. The bacteria belonging to the genera of *Chloroplast*, *Ralstonia*, *Herbaspirillum*, *Muribaculaceae*, and Acinetobacter were found to be dominant and may have contributed to the significant change in color, taste, and volatile compounds during storage [42].

Green teas are also characterized by their theobromine and caffeine content, which are common compounds in cocoa beans. A number of studies on taste-active compounds and the sensory perception of tea infusions were largely done with *Camellia sinesis* with other herbal and floral teas receiving much less attention. Siow et al. (2022) [43] explored an alternative use of cocoa bean hulls from Malaysia, Vietnam, or Venezuela, by processing them into tea, resembling green tea or black tea. They found that cocoa bean tea from Venezuela or Malaysia had higher theobromine and lower caffeine contents than green teas. Moreover, their antioxidant content was similar to those of coffee and black teas. Vietnam beans exhibited strong sweet flavor, Malaysia beans had strong chocolate-like flavor, while Venezuela beans had strong toasty flavor. Two-thirds of panelists indicated that they would purchase cocoa bean hull tea [43]. Therefore, the use of this by-product of cocoa bean production may present an important research opportunity, considering the product is high in antioxidant content.

### 2.4. Oolong Teas

While green teas are unoxidized and black teas are fully oxidized, the processing of oolong teas involves withering, bruising, fixing, rolling, and drying to achieve anywhere between 8% to 85% of oxidization. Therefore, the traditional Chinese oolong teas exhibit a diverse profile of aroma depending on the region of origin, cultivars, and the steps of processing. Wang et al. (2022) [44] obtained 35 sensory descriptors from oolong tea samples collected from fifteen regions of eight countries and constructed the first flavor wheel for Oolong tea. They also reported that the major marker compounds differentiating oolong teas from different regions were predominantly free amino acids, followed by theanine, catechins, caffeine, ester catechins, and tea polyphenols [44]. Feng et al. (2024) [45] further confirmed that significant differences in the levels of theaflavins, flavonoids, flavone glycosides, alkaloids, and pyrrolidones were observed among oolong teas from different regions. Apart from different regions, flavor compounds can also vary greatly between varieties used. Guo et al. (2021) [46] compared fresh leaves and the oolong tea processed from three cultivars, Shuixian, Huangmeigui, and Zimudan, and found that they presented predominantly “green”, “spicy and woody”, and “floral or waxy” odors, respectively. After a full firing process (to stop further oxidation), roasted, cereal-like, burnt, woody, and floral odors were commonly perceived, while high intensities of popcorn-like, creamy, and caramel-like odors were only present in Huangmeigui and Zimudan. Furthermore, 2-ethyl-3,5-dimethylpyrazine (roasted and caramel), linalool (sweet and floral), and *trans-β*-ionone (violet and raspberry floral) were the key aroma compounds found in all three oolong teas, although specific OAV vary among cultivars. Another study focusing on Beauty Tea (aka., Oriental Beauty or Dongfang Beauty, an oolong tea processed from tea leaves damaged by leafhopper *Empoasca onukii*) found that tea samples collected from four out of eight varieties used for making Beauty Tea in Fujian province received similar high scores from sensory evaluations, with methyl salicylate, linalool, geraniol, and phenyl ethanol content changing from variety to variety [47].

Song et al. (2021) [48] discussed the effect of processing steps on oolong tea sensory quality and component compounds in summer leaves collected from the cultivar Bixiangzao. During processing, contents of flavonoids, polyphenols, caffeine, water extracts, and soluble sugars in tea leaves decreased, while the content of free amino acids increased, promoting the gradual decrease in bitterness, astringency, and greenish flavors and the gradual increase in sweet, mellow, and floral and fruity flavors [48]. One key step in processing certain oolong teas is roasting after oxidation. For example, Wuyi rock tea (WRT) and Dongding tea (DDT) undergo robust roasting to gain the characteristic rock flavor or roasted nutty flavor, while High Mountain tea (HMT) from Taiwan and Tieguanyin tea (TGYT) are lightly oxidized without roasting, thus having a floral and creamy flavor. Roasting can significantly impact the volatile profile of oolong teas with the numbers and intensities of heterocyclic compounds in roasted oolong tea being significantly higher than those in unroasted samples [49]. In another study where roasting was used to improve flavor attributes of TGYT, unroasted TGYT exhibited more pronounced fresh and green flavors, while roasted TGYT had stronger pungent and caramel flavors. Flavonoids, glycosides and procyanidins have been found to decrease after roasting and in conjunction with the reduction of bitterness and astringency in these teas [50]. Compounds such as gallic acid increased with roasting and have, therefore, been determined to be a major contributor to the increased sweet aftertastes [50]. Despite the beneficial effects of roasting, higher temperatures (130 °C for 1 h in addition to 105 °C for 5 h) can be detrimental to desirable compounds such as L-theanine, making the determination of optimal roasting temperatures and periods a priority in the production of oolong teas [50].

Dancong tea is another Chinese oolong tea famous for its floral and honey odors but it may vary greatly among harvests and grades. Chen et al. (2022) [51] evaluated seventy Dancong samples, reporting that all of them possessed floral and honey odors, but at different intensities. They identified that indole, (E)-nerolidol, 2-phenyl acetonitrile, and γ-caprolactone were responsible for the floral odor, and hexyl 2-methylbutanoate, (Z)-3-hexenyl pentanoate, (Z)-linalool oxide (pyranoid), (E)-linalool oxide (furanoid), and (Z)-linalool oxide (furanoid) were major contributors for the honey odor. The presence and amount of these components can serve as predictors in the quality control of Dancong oolong teas [51].

WRT produced in the Wuyi Mountain region in China is another popular oolong tea and has been analyzed by several researchers [52,53,54]. Guo et al. (2021) [52] analyzed WRT from withering to full fire processing, and found that floral compounds (linalool, geraniol, nerolidiol, and phytol) gradually decreased until roasting. From roasting to full fire processing, α-farnesene (citrus odor) decreased and benzaldehyde (caramel odor) increased. These chemical changes were perceived by a trained sensory panel who determined that the full fire WRT had strong floral, nutty, roasted, coffee/cocoa-like and burnt odors, and moderate woody and cereal-like odors, which were characteristic of WRT; therefore, a full fire processing is essential to develop this tea. Jiang et al. (2022) [53] compared WRT from roasted and non-roasted leaves and reported higher bitterness and astringency taste in the non-roasted teas. The main differences between non-roasted and roasted teas were the content of flavan-3-ols and gallic acids, where the content of the main galloylated (EGCG, ECG, and GCG) and simple catechins, caffeine, and theobromine in roasted tea were significantly lower than those in non-roasted tea. Roasting temperatures between 80 and 160 °C were best in reducing tannins, which highly influenced bitterness and astringency scores rated by the trained sensory panel. Roasting at 100 °C was a critical point for effectively decreasing the astringent intensity of tea. It is important to note that this study found bitterness and astringency taste scores were not affected by the low threshold flavanol glycosides; therefore, these compounds could be a viable method for lowering the perception of these flavors. Pang et al. (2022) [54] compared quality indexes between four different grades of WRT [gold medal, first prize, second prize, and non-awarding tea] and their chemical compositions. The lower grade teas had lower (but not statistically significant) water extract and caffeine content while the higher-grade teas had higher sugar and polyphenols content. The contents of tea polyphenols, caffeine, EGCG, and EGC were significantly and positively correlated to the total quality scores rated by the sensory panel, and thus can be used to evaluate the grade differences of WRT [54]. Yue et al. (2023) [55] investigated the aroma characteristics of WRTs prepared from 16 varieties of *C. sinensis* considered to be suitable for making oolong tea, and concluded that all 16 WRTs had the “rock” flavor eliciting floral, fruity, and woody odors. The aroma profiles of WRTs were largely characterized by the cultivar-specific volatile compounds. They further reported that β-ionone, geraniol, benzene acetaldehyde, benzoic acid, methyl ester, and indole were predominant floral odor volatiles with high rOAV in all WRT samples [55].

### 2.5. Yellow Teas

Yellow teas are very similar to green teas in terms of processing, with the important distinction that this tea includes a “yellowing” step. During yellowing, tea leaves are stacked into piles and maintained at constant humidity and room temperature, which generates the characteristic yellow color, taste, and aroma profile due to the increase in alcohol and aldehyde compounds [56]. Sheng et al. (2024) [57] investigated the effects of three roasting methods on the aroma quality of large-leaf yellow tea (LYT), and reported that charcoal roasting yielded LYT with rice crust and burnt aroma, while drum roasting yielded the more pronounced floral and corn aroma. In contrast, the electric roasting LYT aroma was more similar to the charcoal roasting. The nine discriminating key aromas were 2,4,5-trimethyloxazole, 1-ethylpyrrole-2-carboxaldehyde, 2-ethyl-3,5-dimethylpyrazine, 3-ethyl-2,5-dimethylpyrazine, linalool, 2, 3-diethyl-5-methylpyrazine, 3, 5-diethyl-2-methylpyrazine, *β*-damascenone, and (*E*)-*β*-ionone (Sheng et al. (2024)). Although charcoal roasting provides the most favorable aromas of LYT, considering the economic and environmental benefits, electric roasting is worth promoting [57].

Liu et al. (2024) [58] investigated the contribution of tea stems to the aroma profile of large-leaf yellow tea (LYT). Using HS-SPME and SBSE in combination with GC–MS, significant differences in volatile compounds between the stems and leaves of LYT were found, with a greater level of pyrazines in stems, and of alcohols, aldehydes, and ketones in leaves. GC-O and OAV analyses confirmed that the elevated pyrazine levels (particularly 2-ethyl-3,5-dimethylpyrazine, 2-ethyl-3,6-dimethylpyrazine, 3,5-diethyl-2-methylpyrazine, and 1-ethyl-1H-pyrrole-2-carbaldehyde) in the stems contributed to the roasty aroma of LYT [58]. This study suggested that retaining the stems and leaves together, both during traditional processing and at the point of sale, is beneficial to the quality characteristics of LYT. Fan et al. (2022) [59] used sensory quantitative descriptive analysis (QDA) and HPLC to determine the changes undergone by tea leaves during the yellowing step extended from 0 to 13 h, and observed a decrease in catechins, flavanol glycosides and caffeine and an increase in certain amino acids, which contributed to the elevated sweet and mellow tastes. The increases in gallic acid, serine, tyrosine, threonine and alanine were closely related to the intensity of the sweet aroma and mellow tastes [59]. This finding provides a basis for further improving the quality of yellow tea.

To shorten the processing time from 2–3 days to 16 h, Wei et al. (2023) [60] developed the optimized yellowing process, with a controlled temperature (34 ± 1 °C) and relative humidity (67% ± 2%) compared to the 15–25 °C and 40–60% of the traditional natural yellowing process. Wei et al. (2024) [61] used GC–MS and GC-O combined with sensomics analysis to identify the key odorants in yellow tea from this optimized process, including dimethyl sulfide, 3-methylbutanal, *β*-ionone, *β*-damascenone, geraniol, phenylacetaldehyde, and linalool, which were mainly characterized by corn-like, sweet, and floral aromas. Among them, *β*-damascenone was the main odorant for sweet aroma enhancement, while *β*-ionone was the main odorant for floral aroma enhancement [61].

### 2.6. White Teas

White tea (WT) is a light fermented tea, originating from the Fujian Province, China. With the increasing worldwide popularity, it is now grown not only in other parts of China but also in other countries [62]. WT is known for its health benefits. As being the least processed type of tea, WT is known for preserving a high level of bioactive phytochemicals from fresh tea leaves. The traditional process for WT does not include stir-frying nor rolling, resulting in the tea with the quality characteristics of “natural shape, apricot-like white color, endoplasmic fragrance, fresh taste” [63]. Withering is the most important processing step during WT production due to the buildup of amino acids.

Zou et al. (2024) [64] evaluated the effect of solar withering (SW) on aroma formation in WT and the underlying mechanism through HS-SPME-GC–MS and transcriptomics. They reported that increases in non-ester fatty acid-derived volatiles and volatile terpenoids in WT subjected to SW mainly contributed to the green, fresh, floral, and fruity odors of the WT, while decreases in amino acid-derived volatiles contributed to sweet, fruity, and floral odors. Wu et al. (2024) [65] compared effects of SW and withering-tank withering (WW) on aroma. They concluded that SW enhanced the floral aroma of white tea, whereas WW enriched its grassy aroma. The former was linked to the geranyl pyrophosphate synthase and alcohol dehydrogenase activity, while the latter was linked to the elevated levels of lipoxygenase and arogenate dehydratase. Mu et al. (2021) [63] used sensory trained panelists and HPLC to determine the effect of the light-emitting diodes (LEDs) light (red, yellow, blue, or green light) used during withering on the aroma compounds. Tea samples under the green LED light had higher polyphenol content, and generated the highest amino acid content under the red LED light. Additionally, the yellow-light-withered samples had the highest water content. Based on sensory evaluation, the red-light group had the highest sensory score, and samples withered without LED had the lowest. These findings indicate that the light source can significantly impact the final white tea produced [63].

Chen et al. (2024) [66] evaluated Zhenghe white tea (ZHWT), Fuding white tea (FDWT), and Jinggu white tea (JGWT) collected from different production years (2022, 2021, 2020, 2019, and 2007) with storage years of 0, 1, 2, 3, and 15 years, respectively. Using nontargeted metabolomics and quantitative analysis, they identified a total of 83 (ZHWT vs. FDWT), 89 (ZHWT vs. JGWT), and 75 (FDWT vs. JGWT) differential compounds, of which amino acids and flavonol/flavone glycosides exhibited the highest number of variations. In terms of storage time, the levels of flavanols, dimeric catechins, and amino acids declined with extended storage, while all *N*-ethyl-2-pyrrolidone-substituted flavanols compounds, caffeine, adenosine monophosphate, and adenosine increased. The initial taste profile, characterized by sweetness, freshness, bitterness, and a slight astringency, transitioned into a sweeter and mellower taste profile. Therefore, the quality of white tea is subject to variations based on its origin and storage duration [66].

**Table 1:** Major chemical and sensory changes of tea quality as affected by several factors *.

Type of Tea	Factors Affecting Tea Quality	Major Chemical or Sensory Changes	Methods Used to Detect Changes	References
Green tea	Increased withering	Increase in dimethyl sulfide, nonanal, isovaleraldehyde, (Z)-3-hexenol, linalool; increased pleasant aroma	GC–MS; trained sensory panelists	[37]
	Increased roasting temperature	Decrease in flavonoids; enhancement of roasty and burnt odor	GC–MS; trained sensory panelists	[38]
	Harvest time	Summer harvest leads to increased L-theanine; increased green tea aroma	GC–MS; trained sensory panelists	[39]
	Basidiomycetes in fermentation	Decrease in (E, E)-2,4,decadienal, geraniol, and (E)-methyl jasmonate; decrease in green tea flavor	GC–MS; trained sensory panelists	[41]
	Storage time	Quality improved or deteriorated by changes in microbial communities	Near-infrared spectroscopy, 16S rDNA sequencing; trained sensory panelists	[42]
Yellow tea	Roasting method	Changes in the predominant aroma of the tea	GC–MS; trained sensory panelists	[57]
	Presence or absence of stems	A higher level of pyrazines in stems; a higher level of alcohols, aldehydes, and ketones in leaves; enhanced roasty aroma	GC–MS, GC-O, OAV	[58]
	Duration of yellowing	Longer yellowing decreased catechins, flavanol glycosides, and caffeine; increased gallic acid, serine, tyrosine, threonine and alanine; enhanced sweet and mellow tastes	HPLC; sensory QDA	[59]
	Increased temperature and relative humidity during yellowing	Increase in β-damascenone and sweet aroma enhancement; β-ionone increased and floral aroma enhancement	GC–MS, GC-O; sensomics analysis	[60,61]
White tea	Solar withering	Increase in non-ester fatty acid-derived volatiles; increased green, fresh, floral, and fruity odors	GC–MS, transcriptomics	[64,65]
	Withering-tank withering	Increase in gerany pyrophosphate synthase and alcohol dehydrogenase activity; enhanced grassy aroma	GC–MS, metabolomics	[65]
	Use of LED lights during withering	Green LED light increased polyphenol content; red LED light increased amino acid content; yellow LED light presented highest water content; samples withered with red LED light were more preferred	HPLC; trained sensory panelists	[63]
	Length of storage	Amino acids, dimeric catechins, and flavonol/flavone glycosides declined with extended storage, N-ethyl-2-pyrrolidone-substituted flavonols, caffeine, adenosinemonophosphate, and adenosine increased; with longer storage taste profile was sweeter and mellower	Metabolomics, LC–MS	[66]
Oolong tea	Growing region	Differences in the presence of free amino acids, theanine, catechins, caffeine, ester catechins, polyphenols, theaflavins, flavonoids, flavone glycosides, alkaloids, and pyrrolidones	QDA, UPLC-QTOF-MS; trained sensory panelists	[44,45]
	Varieties	Varieties used can predominantly be associated with either green, spicy and woody, and floral/waxy odors; common compounds among varieties include 2-ethyl-3,5-dimethylpyrazine (roasted and caramel aroma), linalool (sweet and floral aroma), and trans-β-ionone (violet and raspberry floral aroma)	GC–MS, GC–IMS, OAV; trained sensory panelists	[46,47,55]
	Processing	Decrease in flavonoids, polyphenols, caffeine, water extracts, and soluble sugars; increase in free amino acids; increase in sweet, mellow, and floral and fruity flavors	GC–MS; trained sensory panelists	[48]
	Roasting	Increase in heterocyclic compounds; decrease in flavonoids, glycosides, and procyanidins; stronger pungent and caramel flavors when roasted	GC–MS, HPLC, UHPLC; trained sensory panelists	[49,50,52,53]
	Harvest and grade	Indole, (E)-nerolidol, 2-phenyl acetonitrile, and γ-caprolactone were responsible for the floral odor; hexyl 2-methylbutanoate, (Z)-3-hexenyl pentanoate, (Z)-linalool oxide (pyranoid), (E)-linalool oxide (furanoid), and (Z)-linalool oxide were responsible for honey odor	GC–MS; trained sensory panelists	[51,54]
Black tea	Grade	Lower grades had increased sweet aroma, higher grades had increased keemum aroma; higher grades had higher geraniol, linalool, and methyl salicylate	GC–MS; Sensory QDA	[17]
	Fermentation time	Three-hour fermentation retained the highest content of catechins	HPLC, GC–MS, OAV; trained sensory panelists	[18]
	Fermentation with *Cordyceps militaris*	Reduced polyphenol, flavonoids, and free amino acids	LC–MS, GC–MS metabolomics; trained sensory panelists	[19]
	Drying method	Hot air dried teas scored higher than those dried with hot roller dryer; hot air drying generated sweet and flowery flavors; hot roller drying generated fruity flavors	GC–MS, LC–MS; trained sensory panelists	[20,21]
	Withering	Sun-withered samples were more bitter and astringent; electric tongue determined sun-withered samples to be sweeter and more astringent due to higher glucose and sugar breakdown; longer withering increased sugar and amino acids	Electric tongue, HPLC; trained sensory panelists	[22,23]
	Length of storage	Longer storage reduced epicatechin and epigallocatechin gallate; caffeine and theobromine levels were not affected	HPLC, LC-TMS; trained sensory panelists	[24]
	Region	Samples from China (higher linalool and benzeneacetaldehyde) and India (higher geraniol, phenylethyl, and linalool) had higher intensities of sweet and floral aromas; Sri Lankan samples had higher methyl salicylate and peppermint aroma	GC–MS, GC-E-Nose; trained sensory panelists	[26,27]
	High-altitude growing area	(E)-2-octenal, cis-3-hexenyl hexanoate, (E)-2-hexenyl hexanoate, linalool, d-cadinene, octanal, (E,E)-2,4-heptadienal, epoxy linalool, methyl salicylate, 2,6-bis(1,1-dimethylethyl)-2,5-Cyclohexadiene-1,4-dione, ethyl 2-(5-methyl-5-vinyltetrahydrofuran-2-yl)propan-2-yl carbonate, and hexadecane compounds could distinguish between high and low altitude teas	GC–MS, GC-O	[28]
Dark tea	Post-fermentation	Sweetness remained stable during post-fermentation; six days onwards post-fermentation astringency, bitterness, and sourness decreased; nine days onwards post-fermentation mellowness increased	UPLC-Q-TOF/MS, LC–MS; trained sensory panelists	[33]
	Fermentation	*Aspergillus*, *Candida*, *unclassified-o-Hypocreales*, *unclassified-o-Saccharomycetales*, and *Wal-lemia*, and the bacterial genus *Klebsiella* were essential for fungal aroma formation; tank fermentation led to lower astringency, bitterness, and sourness	GC-Q-TOF-MS, GC-O, AEDA, UPLC-Q-TOF/MS, LC–MS; trained sensory panelists	[32,33,34]
	Length of storage	Taste changes at 7-years storage; after 25–35 years astringency decreased; quercetin, quercetin 3-O-glucuronide, glycine, aspartic acid, alanine, serine, arginine, threonine, tyrosine, theanine, γ-aminobutyric acid (GABA), and isoleucine decreased with time; quercetin 3-O-rutinoside increased with time	Metabolomics; trained sensory panelists	[35]

* Based on publications from 2021 to 2024.

## 3. Current Analytical and Sensory Evaluation Methods for Tea

**Figure 2:**
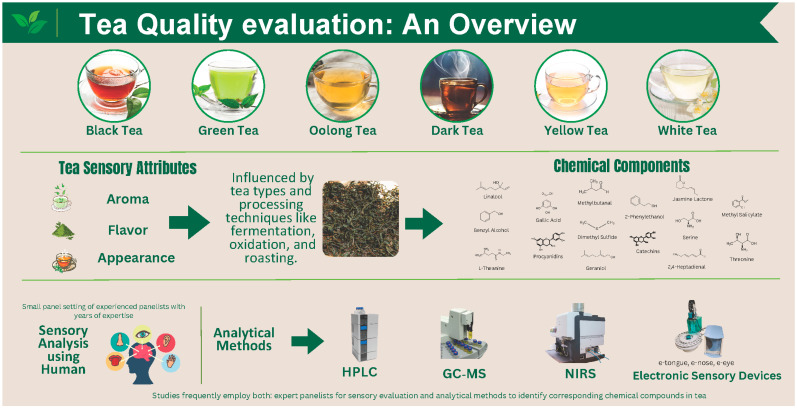
Analytical methods and sensory evaluation used to evaluate quality of teas.

The traditional methods for classifying tea varietals and rating tea characteristics include human and instrumental sensory investigation (Figure 2) with the three most popular methods being the electronic tongue method, the sensory assessment method, and the gustatory test method. The electronic tongue technique seems to be the most promising and has been used as an auxiliary device combined with sensory evaluation to obtain more specific and quantifiable results. [67]. Tea specialists employ the aroma, liquor color, texture mouthfeel, and morphological characteristics as determining elements to differentiate the tea quality grade. This procedure, however, may lead to inconsistent test results when used with various groups of testers with different trainings and years of experiences [68]. Moreover, it is challenging for customers to comprehend the study because it calls for experienced panel assessors to have their own opinions on the distinct tea sensory features [69]. Due to the difficulty and length of time involved in this sensory method, mass detection and identification are not practical. Furthermore, it is challenging for tea researchers from many nations to create standardized terminology that can uniformly describe the flavor of tea infusion [68]. It takes skilled and knowledgeable evaluators to produce precise and accurate sensory results, and training a professional evaluator can be expensive in terms of time, money, human subjects, and material resources.

The outcomes of the examination are significantly influenced by the evaluator’s health, subjective judgment, changes in sensory sensitivity that occur throughout the evaluation, and other ambient elements in the test room (such as light, sound, and color). Therefore, it is not advised to solely employ sensory evaluation when a lot of tea samples need to be examined. However, the primary method of evaluation in the tea industry, despite these drawbacks, is still sensory assessment because of its exceptional discriminatory capacity [67].

Analytical methods such as spectroscopy, machine vision systems, and hyperspectral imaging have recently replaced traditional analytical methods to detect and monitor the chemical compounds responsible for the flavor, odor, and color of tea and its products. Additionally, numerous analytical methods have been recently developed for determining the quality of tea, including capillary electrophoresis (CE), plasma atomic emission spectrometry, high-performance liquid chromatography (HPLC), gas chromatography–mass spectrometry (GC–MS), and ultraperformance liquid chromatography (UPLC). These techniques are highly sensitive and selective, but they are also very expensive and call for several pretreatment processes for sample preparation [69,70]. A summary of the applications, advantages, and drawbacks of commonly used methods is shown in Table 2.

Recently, special-grade teas have been categorized using near-infrared spectroscopy (NIRS), which measures bioactive substances with wavelengths between 780 and 2526 nm. This mechanism operates based on how the hydrogen-containing groups—such as C-H, N-H, and O-H—respond to NIR light, hence tea polyphenols, catechins, theaflavins, caffeine, and chlorophyll can all be predicted with the help of NIRS [70]. NIRS has also been used to evaluate the quality of green teas with promising results indicating that it can be used to accurately predict physical characteristics and sensory qualities [71].

**Table 2:** Summary of analytical and sensory evaluation methods commonly used for tea quality evaluation and their advantages and drawbacks.

Method	Tea Quality Measured	Advantages	Drawbacks	Some Selected References
Human Sensory Analysis (including GC-O)	Sensory attributes: appearance of dry tea; taste, aroma, texture mouthfeel, and color of tea infusions	Direct assessment of sensory attributes	Subjective, requires trained panelists, inconsistent results, sensory fatigue	[8,17,22,26,27,28,32,39,40,41,51,59,63]
Gas Chromatography–Mass Spectrometry (GC–MS)	Volatile aroma compounds	Accurate identification and quantification of volatile compounds	Costly, requires sample preparation and pretreatment	[12,17,22,24,26,27,28,32,44,46,52,53,54,61,64,66]
High-Performance Liquid Chromatography (HPLC)	Non-volatile compounds, catechins, caffeine	Precise quantification of compounds	Costly, complex sample preparation needed	[7,22,24,59,63,69,70]
Ultraperformance Liquid Chromatography (UPLC)	Non-volatile compounds (similar to HPLC but with enhanced capabilities)	High sensitivity and selectivity	Requires extensive preparation and expertise	[33,40,70]
Capillary Electrophoresis (CE)	Ionic species in tea	Effective for separating ionic species, inexpensive	Limited to charged molecules, complex operation	[70,72]
Plasma Atomic Emission Spectrometry	Elemental composition	Broad element detection capability	High cost, complex sample handling	[70]
Near-Infrared Spectroscopy (NIRS)	Prediction of polyphenols, catechins, caffeine	Rapid, non-destructive, no sample preparation	Limited to certain compounds, may need calibration	[29,30,31,70,71]
Electronic Nose/Tongue	General aroma, basic taste and flavor profile	Objective, fast, suitable for large sample analysis	Limited by sensitivity to minor compound variations, affected by external conditions, no detailed identification information	[27,67,69,70]

It is very common that researchers combine various techniques to analyze the various aroma compounds of teas rather than using a single method. The most popular techniques for tea aroma analysis mostly involve the headspace solid phase microextraction (HS-SPME), the simultaneous distillation extraction (SDE), and the stir bar sorptive extraction (SBSE), which are used to extract the scent of tea. After the extraction of volatile substances, the analysis can be done by a combination of methods such as gas chromatography–mass spectrometry (GC–MS), gas chromatography-olfactometry (GC-O), relative odor activity value (rOAV), and sensory analysis using a trained sensory panel to investigate the aroma components [14]. As mentioned above, sensory evaluation performed by human subjects is used frequently for tea quality evaluation. The safety of the sensory panelists are top priorities. Herbicides may be used in tea cultivation which raises concerns about human health and environmental hazards. Nguyen et al. (2022) [72] applied a new approach using a two-channel capillary electrophoresis system to simultaneously analyze several herbicides and their metabolite compounds.

The real-time polymerase chain reaction (PCR) has also been used as part of the tea quality evaluation. In a study with oolong tea, the aroma formation during the processing of Jinxuan and Qingxin tea was analyzed using GC–MS while enzymatic activity which affected the aroma production during processing was measured by transcription analysis using real-time PCR [11]. The analytical methods can not only help determine the aroma characteristics and quality of tea but also help determine the origins of the teas. The chemometric analysis and headspace GC–MS were used to determine the origins of 306 black tea samples from three different countries and 10 production regions. These teas from various sources were distinguished by the differences in the concentration of key volatile chemicals such alcohols, aldehydes, ketones, and esters. In this case, GC–MS was used to produce a full-spectrum of aroma data, and data analysis using the k-Nearest Neighbor model was used to eliminate complex sample data processing and cut down on the time needed for spectral analysis and, therefore, enabled quick location identification of tea samples from different regions [73]. On the other hand, some types of teas may be geographically identified by sensory experts; however, it is susceptible to individual variability and is subjective, inconsistent, and unpredictable.

Although some analytical methods are highly accurate and sensitive, they are expensive; therefore, it is crucial to search for reliable and quick substitutes in contemporary tea enterprises. When compared to the traditional methods, the use of an electronic nose, tongue, or eye (e-nose, e-tongue, or e-eye) can help determine the quality of tea [70]. Accurate food analysis findings are now rapidly achievable because of the quick development of multi-sensor and electrical technology. The color, gas, and liquid sensors that make up the e-eye, e-nose, and e-tongue systems replicate the human visual, olfactory, and gustatory systems. With these technologies, there is minimal or no sample pretreatment [69]. The e-tongue device is used to assess the taste of tea by simulating the human palate for the taste categorization and analysis of different chemicals in liquid-phase samples. It transforms chemical signals into electrical signals, and then displays the detection results visually. The e-tongue has clear advantages such as a wider detection range, faster detection speed, the ease of use, steady accuracy and sensitivity with increasing evaluation duration, and more objective results. However, there are certain drawbacks. For instance, minute component variations in the test samples will affect how the sensory array responds, and external influences like temperature and other ambient conditions or surface flaws resulting from cross-contamination might also affect the sensitivity of the sensors [67].

## 4. New Trending Methods of Tea Quality and Tea Alternatives

Technological advancements in tea processing, chemical analysis, and sensory evaluation have played a significant role in expanding the marketability of tea on a global scale. One such method of analysis involves the identification and evaluation of the volatile flavor compounds found in different cultivars of black tea around the world [74]. Once isolated and identified, these flavor components give insights on which flavors are highly sought after in which regions of the world, thus allowing producers to target those regions specifically with the teas that will be well received by the target market.

Though black tea is widely consumed globally, green tea is currently the most consumed of all of the tea varieties and stands as one of the most popular beverages, after water, in the world [74]. Therefore, it is imperative that new methods of treatment be developed and researched to improve the quality of green tea. One such treatment is the drying method, which currently serves as an important final step in the process of producing black tea; baking tea removes undesirable aromas, reduces water content and provides more shelf-stability, and reduces bitterness and astringency in the black tea [74]. By introducing this treatment to green tea, Wang et al. (2022) [74] aimed to enhance the flavor compounds found in green tea in ways that reflect the benefits of baking in black tea. Green tea has also been enriched with selenium due to its possible health benefits; however, selenium has been found to have a significant impact on the content of the aromatic compound of this product. High selenium contents led to the decrease in non-gallated catechins and increase in gallated catechins [75]. The results from this study may warrant more research to better understand the aroma chemistry of selenium-enriched green tea and to increase its health benefits without affecting sensory characteristics.

In addition to utilizing these new methods on *Camellia sinensis*-based tea products, it has become increasingly vital that more research be conducted on herbal tisane to help develop and market new products aimed towards specific regions. The steeping of dried herbs and flowers can be found throughout countless cultures, which serves as a vital caffeine-free alternative to traditional tea and warrants more research. One such example is the evaluation of the taste preferences of different flavored indigenous herbs containing dried corn silk powder [76]. These indigenous herbs were identified as underutilized in tisanes despite their huge dietary potential [76]. The study results showed high acceptability in terms of all sensory parameters, which indicates that utilizing herbs indigenous to specific regions in both true tea and herbal tisane blends could improve the overall taste and marketability of products within said regions.

## 5. Conclusions and Future Studies

The chemical composition, including volatile and non-volatile compounds and some bioactive compounds, contributes to the diverse and distinct characteristics of different tea infusions. Analytical techniques such as GC–MS and HPLC play a crucial role in determining this composition by identifying and quantifying specific compounds, while providing valuable data that could be correlated to sensory attributes and their intensities as measured primarily by a trained sensory panel. Specifically, aroma has been influenced by different compounds, many of which vary by tea varieties and the processing methods of the tea leaves. Human sensory evaluations of tea infusions continue to be a major tool for evaluating tea aroma and flavor characteristics; however, the high level of training required for panelists may hinder its usage for identification (lexicon development) and intensity quantification. This limitation highlights the emerging trends of using e-nose and e-tongue for sensory evaluations. However, consumer preferences, acceptance, and purchase intent must be evaluated by consumers.

This current review provided an overview of various key chemical components associated with sensory characteristics of the six most consumed teas (black, dark, green, oolong, yellow, and white teas) and discussed different identification and quantification methods of selected chemical compositions based on publications between 2021 and 2024. During processing, volatile substances are altered by enzymatic and thermophysical processes, and account for the majority of the final scent, whereas non-volatile components are inherent to the fresh leaves contributing relatively less to aroma. In general, the contents of total volatiles, aliphatics, aromatics, and terpenoids increased with the degree of fermentation during tea processing, whereas jasmine lactone and indole were found in a large quantity in oolong teas. In terms of taste, sweet and umami tastes are usually well-accepted for consumers, whereas the bitter and astringent tastes are often undesired, but they constitute the holistic sensory perceptions of Camellia teas. In the tea infusion, flavonol-*O*-glycosides, tannins, and galloylated catechins are the main astringent compounds; caffeine and non-galloylated catechins are responsible for bitterness. A sweet aftertaste is a unique perception of green tea, which is attributed to the hydrolysis of galloylated catechins. L-theanine, succinic acid, gallic acid, and theogallin contribute to the umami taste.

Moving forward, the evaluation of tea infusions would require a broader understanding of the chemical and sensory characteristics of specific infusions to improve tea leaf processing techniques and meet the demand from consumers and market potential for high quality and healthy teas. Measuring tea quality indicators as a function of variety and genetic diversity, regions, processing methods, or production practices (e.g., treatment effects of fertilization, shading, and harvesting methods) is critical for the quality of a final tea product. The findings will help inform tea growers and processors about the preferred aroma/flavor, overall quality, and chemical composition, which will enable them to optimize their production methods and correlate consumer preferences with chemical properties of tea varieties. This could enhance the marketability of teas and provide detailed conditions for optimizing processing steps to obtain final tea products with desirable quality. In order to do so, future research is needed to establish a combination of advanced analytical and sensory methods to be used to improve aroma/flavor quality, identify new functional chemical components, and elucidate the interactions between functional components.

In this review, most studies used some forms of a trained sensory descriptive panel to evaluate the sensory profile of tea infusions. However, it is possible to use a consumer panel via the Check-All-That-Apply method to come up with a sensory profile of tea infusions, hence, more research is needed in this area. Different multivariate statistical methods, bioinformatics and artificial intelligence algorithms can be employed to identify the key chemical indices closely associated with the sensory quality of fresh and dry teas. Artificial intelligence algorithms show great application potential for classifying tea products with different sensory qualities; hence, they may be used to predict the sensory qualities of tea based on the discriminating chemical indices. Another needed research area is rapid discrimination technologies for the comprehensive evaluation of tea product quality, during grading and quality control. Gas chromatography–ion mobility spectroscopy (GC–IMS) is a novel detection technology as it combines the advantages of GC and IMS, respectively, in terms of separation capacity for a complex aroma matrix, and the high sensitivity and rapid response for volatile compounds. A combination of different analytical methods such as GC–IMS, NIR spectroscopy, e-eye, e-nose, and e-tongue technology to comprehensively evaluate color, appearance, aroma, and taste of tea leaves through a cross-sensor multimodal fusion system is needed.

## Data Availability

No new data were created or analyzed in this study. Data sharing is not applicable to this article.

## Abbreviations

HPLC	high-performance liquid chromatography
GC–MS	gas chromatography–mass spectrometry
CE	capillary electrophoresis
UPLC	ultraperformance liquid chromatography
NIRs	near-infrared spectroscopy
SBSE	stir bar sorptive extraction
GC-O	gas chromatography-olfactometry
OAV	odor activity value
KBT	Keemun black tea
QDA	Quantitative Descriptive Analysis
LC	liquid chromatography
TMS	tandem mass spectrometry
EGCG	epigallocatechin gallate
HS-SPME	headspace solid-phase microextraction
DBT	Dianhong black tea
GC-E-Nose	gas chromatography-electronic nose
CVS	computer vision system
PCA	principal component analysis
SVM	support vector machine
FBT	Fu brick tea
Q-TOF/MS	quadrupole-time of flight mass spectrometer
AEDA	aroma extraction dilution analysis
AT	An tea
GABA	γ-aminobutyric acid
TAV	taste activity value
WRT	Wuyi rock tea
DDT	Dongding tea
HMT	High Mountain tea
TGYT	Tieguanyin tea
ECG	Epicatechin-3-gallate
GCG	gallocatechin gallate
EGC	epigallocatechin
rOAV	relative odor activity value
LYT	large-leaf yellow tea
WT	white tea
SW	solar withering
WW	withering-tank withering
LED	light-emitting diode
ZHWT	Zhenghe white tea
FDWT	Fuding white tea
JGWT	Jinggu white tea
SDE	simultaneous distillation extraction
PCR	polymerase chain reaction
IMS	ion mobility spectrometry

## References

[1] Nair K.P., Nair K.P. (2021). Tea (*Camellia sinensis* L.). Tree Crops: Harvesting Cash from the World’s Important Cash Crops.

[2] Statista. Tea—United States. https://www.statista.com/outlook/cmo/hot-drinks/tea/united-states#methodology.

[3] Liang S., Granato D., Zou C., Gao Y., Zhu Y., Zhang L., Yin J.-F., Zhou W., Xu Y.-Q. (2021). Processing technologies for manufacturing tea beverages: From traditional to advanced hybrid processes. Trends Food Sci. Technol..

[4] Lin F.J., Wei X.L., Liu H.Y., Li H., Xia Y., Wu D.T., Zhang P.Z., Gandhi G.R., Li H.B., Gan R.Y. (2021). State-of-the-art review of dark tea: From chemistry to health benefits. Trends Food Sci. Technol..

[5] Samanta S. (2022). Potential bioactive components and health promotional benefits of tea (*Camellia sinensis*). J. Am. Nutr. Assoc..

[6] Tea Association USA (2023). State of the U.S. Tea Industry, Review of 2023 and A Look Forward into 2024. https://teausa.org//teausa/images/USTA_State_of_the_Industry_2023-2024.pdf.

[7] Zhang X., Du X., Li Y.Z., Nie C.N., Wang C.M., Bian J.L., Luo F. (2022). Are organic acids really related to the sour taste difference between Chinese black tea and green tea?. Food Sci. Nutr..

[8] Su T.C., Yang M.J., Huang H.H., Kuo C.C., Chen L.Y. (2021). Using sensory wheels to characterize consumers’ perception for authentication of Taiwan specialty teas. Foods.

[9] Xiao M., Liu S., Jin H., Xiao M., Wang H., Zhang H., Dai Q. (2022). Evaluating freshness loss of green tea with Q10 Method and Weibull hazard analysis under accelerated shelf life testing. J. Chem..

[10] Zhang L., Cao Q.Q., Granato D., Xu Y.Q., Ho C.T. (2020). Association between chemistry and taste of tea: A review. Trends Food Sci. Technol..

[11] Liu H., Li S., Zhong Y., Lan S., Brennan C.S., Wang Q., Ma L. (2021). Study of aroma compound formations and transformations during Jinxuan and Qingxin oolong tea processing. Int. J. Food Sci. Technol..

[12] Yang P., Song H., Lin Y., Guo T., Wang L., Xu Y. (2021). Differences of characteristic aroma compounds in Rougui tea leaves with different roasting temperature analyzed by switchable GC-O-MS and GC × GC-O-MS and sensory evaluation. Food Funct..

[13] Wu S., Gu D., Chen Y., Wang F., Qian J., Zeng L., Tang J., Yan Y., Chen C., Li J. (2023). Variations in oolong tea key characrieristic floral aroma compound contents among tea (*Camellia sinensis*) germplasms exposed to postharvest stress. Postharvest Biol. Technol..

[14] Zhu J., Zhu Y., Wang K., Niu Y., Xiao Z. (2021). Characterization of key aroma compounds and enantiomer distribution in Longjing tea. Food Chem..

[15] Zeng L., Fu Y.-Q., Liu Y.-Y., Huang J.-S., Chen J.-X., Yin J.-F., Jin S., Sun W.-J., Xu Y.-Q. (2023). Comparative analysis of different grades of Tieguanyin oolong tea based on metabolomics and sensory evaluation. Lwt.

[16] Chen Z., Li Z., Zhao Y., Zhu M., Li J., Wang K. (2024). A meta-analysis of dynamic changes of key aroma compounds during black tea processing. Food Biosci..

[17] Su D., He J.J., Zhou Y.Z., Li Y.L., Zhou H.J. (2022). Aroma effects of key volatile compounds in Keemun black tea at different grades: HS-SPME-GC-MS, sensory evaluation, and chemometrics. Food Chem..

[18] Wang H., Shen S., Wang J., Jiang Y., Li J., Yang Y., Hua J., Yuan H. (2022). Novel insight into the effect of fermentation time on quality of Yunnan Congou black tea. Lwt.

[19] Zhang Y.Y., Zhang P., Le M.M., Qi Y., Yang Z., Hu F.L., Ling T.J., Bao G.H. (2023). Improving flavor of summer Keemun black tea by solid-state fermentation using Cordyceps militaris revealed by LC/MS-based metabolomics and GC/MS analysis. Food Chem..

[20] Ye F., Qiao X., Gui A., Wang S., Liu P., Wang X., Teng J., Zheng L., Feng L., Han H. (2021). Metabolomics provides a novel interpretation of the changes in main compounds during black tea processing through different drying methods. Molecules.

[21] Su S., Long P., Zhang Q., Wen M., Han Z., Zhou F., Ke J., Wan X., Ho C.T., Zhang L. (2024). Chemical, sensory and biological variations of black tea under different drying temperatures. Food Chem..

[22] Huang W., Lu G., Deng W.-W., Ning J. (2022). Effects of different withering methods on the taste of Keemun black tea. Lwt.

[23] Ntezimana B., Li Y., He C., Yu X., Zhou J., Chen Y., Yu Z., Ni D. (2021). Different Withering times affect sensory qualities, chemical components, and nutritional characteristics of black tea. Foods.

[24] Huang A., Jiang Z., Tao M., Wen M., Xiao Z., Zhang L., Zha M., Chen J., Liu Z., Zhang L. (2021). Targeted and nontargeted metabolomics analysis for determining the effect of storage time on the metabolites and taste quality of keemun black tea. Food Chem..

[25] Wen M., Han Z., Cui Y., Ho C.T., Wan X., Zhang L. (2022). Identification of 4-O-p-coumaroylquinic acid as astringent compound of Keemun black tea by efficient integrated approaches of mass spectrometry, turbidity analysis and sensory evaluation. Food Chem..

[26] Wang Q., Qin D., Huang G., Jiang X., Fang K., Wang Q., Ni E., Li B., Pan C., Li H. (2022). Identification and characterization of the key volatile flavor compounds in black teas from distinct regions worldwide. J. Food Sci..

[27] Chen J., Yang Y., Deng Y., Liu Z., Xie J., Shen S., Yuan H., Jiang Y. (2022). Aroma quality evaluation of Dianhong black tea infusions by the combination of rapid gas phase electronic nose and multivariate statistical analysis. Lwt.

[28] Wang B., Chen H., Qu F., Song Y., Di T., Wang P., Zhang X. (2021). Identification of aroma-active components in black teas produced by six Chinese tea cultivars in high-latitude region by GC–MS and GC–O analysis. Eur. Food Res. Technol..

[29] Jin G., Wang Y.J., Li M., Li T., Huang W.J., Li L., Deng W.W., Ning J. (2021). Rapid and real-time detection of black tea fermentation quality by using an inexpensive data fusion system. Food Chem..

[30] Li L., Wang Y., Jin S., Li M., Chen Q., Ning J., Zhang Z. (2021). Evaluation of black tea by using smartphone imaging coupled with micro-near-infrared spectrometer. Spectrochim. Acta A Mol. Biomol. Spectrosc..

[31] Wang Y.-J., Li T.-H., Li L.-Q., Ning J.-M., Zhang Z.-Z. (2021). Evaluating taste-related attributes of black tea by micro-NIRS. J. Food Eng..

[32] Zhao R., Yao H., Hou Z., Zhou Q., Zhao M., Wu C., Zhang L., Xu C., Su H. (2024). Sensomics-assisted analysis unravels the formation of the Fungus Aroma of Fu Brick Tea. Food Chem..

[33] Li Q., Jin Y., Jiang R., Xu Y., Zhang Y., Luo Y., Huang J., Wang K., Liu Z. (2021). Dynamic changes in the metabolite profile and taste characteristics of Fu brick tea during the manufacturing process. Food Chem..

[34] Wang H., Teng J., Huang L., Wei B., Xia N. (2023). Determination of the variations in the metabolic profile and sensory quality of Liupao tea during fermentation through UHPLC-HR-MS metabolomics. Food Chem..

[35] Shen S., Huang J., Li T., Wei Y., Xu S., Wang Y., Ning J. (2022). Untargeted and targeted metabolomics reveals potential marker compounds of an tea during storage. Lwt.

[36] Wang L.F., Kim D.M., Lee C.Y. (2000). Effects of heat processing and storage on flavanols and sensory qualities of green tea beverage. J. Agric. Food Chem..

[37] Qin M., Zhou J., Luo Q., Zhu J., Yu Z., Zhang D., Ni D., Chen Y. (2024). The key aroma components of steamed green tea decoded by sensomics and their changes under different withering degree. Food Chem..

[38] Zhu Y.M., Dong J.J., Jin J., Liu J.H., Zheng X.Q., Lu J.L., Liang Y.R., Ye J.H. (2021). Roasting process shaping the chemical profile of roasted green tea and the association with aroma features. Food Chem..

[39] Guo X., Ho C.T., Schwab W., Wan X. (2021). Aroma profiles of green tea made with fresh tea leaves plucked in summer. Food Chem..

[40] Han Z., Wen M., Zhang H., Zhang L., Wan X., Ho C.T. (2022). LC-MS based metabolomics and sensory evaluation reveal the critical compounds of different grades of Huangshan Maofeng green tea. Food Chem..

[41] Rigling M., Yadav M., Yagishita M., Nedele A.-K., Sun J., Zhang Y. (2021). Biosynthesis of pleasant aroma by enokitake (*Flammulina velutipes*) with a potential use in a novel tea drink. Lwt.

[42] Liu H., Zhuang S., Gu Y., Shen Y., Zhang W., Ma L., Xiao G., Wang Q., Zhong Y. (2023). Effect of storage time on the volatile compounds and taste quality of Meixian green tea. Lwt.

[43] Siow C.S., Chan E.W.C., Wong C.W., Ng C.W. (2022). Antioxidant and sensory evaluation of cocoa (*Theobroma cacao* L.) tea formulated with cocoa bean hull of different origins. Future Foods.

[44] Wang Z., Gan S., Sun W., Chen Z. (2022). Quality characteristics of oolong tea products in different regions and the contribution of thirteen phytochemical components to its taste. Horticulturae.

[45] Feng X., Wang H., Yu Y., Zhu Y., Ma J., Liu Z., Ni L., Lin C.C., Wang K., Liu Y. (2024). Exploration of the flavor diversity of oolong teas: A comprehensive analysis using metabolomics, quantification techniques, and sensory evaluation. Food Res. Int..

[46] Guo X., Schwab W., Ho C.T., Song C., Wan X. (2021). Characterization of the aroma profiles of oolong tea made from three tea cultivars by both GC-MS and GC-IMS. Food Chem..

[47] Zhuang M., Li P., Tu Y., Li P., Yan J., He C., Chen M., Jin S. (2022). Quality evaluation of beauty tea produced by different tea varieties. Chin. J. Trop. Crops.

[48] Song J., He J., Ou Y., Jiang P., Bo J., Gong L., Xiao L. (2021). Dynamic changes in quality and composition of oolong Tea made with fresh Bixiangzao summer tea leaves during processing. Mod. Food Sci. Technol..

[49] Wang D., Liu Z., Chen W., Lan X., Zhan S., Sun Y., Su W., Lin C.C., Ni L. (2023). Comparative study of the volatile fingerprints of roasted and unroasted oolong tea by sensory profiling and HS-SPME-GC-MS. Curr. Res. Food Sci..

[50] Cao Q.Q., Fu Y.Q., Wang J.Q., Zhang L., Wang F., Yin J.F., Xu Y.Q. (2021). Sensory and chemical characteristics of Tieguanyin oolong tea after roasting. Food Chem. X.

[51] Chen W., Hu D., Miao A., Qiu G., Qiao X., Xia H., Ma C. (2022). Understanding the aroma diversity of Dancong tea (*Camellia sinensis*) from the floral and honey odors: Relationship between volatile compounds and sensory characteristics by chemometrics. Food Control.

[52] Guo X., Ho C.T., Wan X., Zhu H., Liu Q., Wen Z. (2021). Changes of volatile compounds and odor profiles in Wuyi rock tea during processing. Food Chem..

[53] Jiang Z., Han Z., Wen M., Ho C.-T., Wu Y., Wang Y., Xu N., Xie Z., Zhang J., Zhang L. (2022). Comprehensive comparison on the chemical metabolites and taste evaluation of tea after roasting using untargeted and pseudotargeted metabolomics. Food Sci. Hum. Wellness.

[54] Pang X., Chen F., Liu G., Zhang Q., Ye J., Lei W., Jia X., He H. (2022). Comparative analysis on the quality of Wuyi Rougui (*Camellia sinensis*) tea with different grades. Food Sci. Technol..

[55] Yue C., Cao H., Zhang S., Hao Z., Wu Z., Luo L., Zeng L. (2023). Aroma characteristics of Wuyi rock tea prepared from 16 different tea plant varieties. Food Chem. X.

[56] Dong R., Sheng X., Xie Q., Huang X., Yan F., Liu S. (2023). Aroma formation and transformation during sealed yellowing process of Pingyang yellow tea. Food Res. Int. J..

[57] Sheng C., Lu M., Liu Q., Zhou H., Xiong Z., Li T., Wei Y., Zhang J., Ke H., Wu Y. (2024). Differences in the aroma quality of large-leaf yellow tea subjected to different roasting methods. Lwt.

[58] Liu Q., Huang W., Sheng C., Wu Y., Lu M., Li T., Zhang J., Wei Y., Wang Y., Ning J. (2024). Contribution of tea stems to large-leaf yellow tea aroma. Food Chem..

[59] Fan F.Y., Zhou S.J., Qian H., Zong B.Z., Huang C.S., Zhu R.L., Guo H.W., Gong S.Y. (2022). Effect of yellowing duration on the chemical profile of yellow tea and the associations with sensory traits. Molecules.

[60] Wei Y., Yin X., Zhao M., Zhang J., Li T., Zhang Y., Wang Y., Ning J. (2023). Metabolomics analysis reveals the mechanism underlying the improvement in the color and taste of yellow tea after optimized yellowing. Food Chem..

[61] Wei Y., Zhang J., Li T., Zhao M., Song Z., Wang Y., Ning J. (2024). GC–MS, GC–O, and sensomics analysis reveals the key odorants underlying the improvement of yellow tea aroma after optimized yellowing. Food Chem..

[62] Guo A.Q., Feng H.F., Jing P., Lan Y., Cao X.N. (2024). White tea: A review on composition characteristics, extraction techniques, and application potentials. J. Tea Sci. Res..

[63] Mu L., Li T., Tang J., Liu L., Wang R. (2021). Effects of LED light withering on the quality of white tea. Proceedings of the IOP Conference Series: Earth and Environmental Science.

[64] Zou L., Sheng C., Xia D., Zhang J., Wei Y., Ning J. (2024). Mechanism of aroma formation in white tea treated with solar withering. Food Res. Int..

[65] Wu H., Sheng C., Lu M., Ke H., Li T., Wei Y., Shen S., Yin X., Lu C., Wang Y. (2024). Identification of the causes of aroma differences in white tea under different withering methods by targeted metabolomics. Food Biosci..

[66] Chen Z., Dai W., Xiong M., Gao J., Zhou H., Chen D., Li Y. (2024). Metabolomics investigation of the chemical variations in white teas with different producing areas and storage durations. Food Chem. X.

[67] Deng S., Zhang G., Olayemi Aluko O., Mo Z., Mao J., Zhang H., Liu X., Ma M., Wang Q., Liu H. (2022). Bitter and astringent substances in green tea: Composition, human perception mechanisms, evaluation methods and factors influencing their formation. Food Res. Int. J..

[68] Li M., Pan T., Chen Q. (2021). Estimation of tea quality grade using statistical identification of key variables. Food Control.

[69] Kaushal S., Nayi P., Rahadian D., Chen H.-H. (2022). Applications of electronic nose coupled with statistical and intelligent pattern recognition techniques for monitoring tea quality: A review. Agriculture.

[70] Gharibzahedi S.M.T., Barba F.J., Zhou J., Wang M., Altintas Z. (2022). Electronic sensor technologies in monitoring quality of tea: A review. Biosensors.

[71] Zuo Y., Tan G., Xiang D., Chen L., Wang J., Zhang S., Bai Z., Wu Q. (2021). Development of a novel green tea quality roadmap and the complex sensory-associated characteristics exploration using rapid near-infrared spectroscopy technology. Spectrochim. Acta A Mol. Biomol. Spectrosc..

[72] Nguyen M.H., Nguyen T.D., Vu M.T., Duong H.A., Pham H.V. (2022). Determination of glyphosate, glufosinate, and their major metabolites in tea infusions by dual-channel capillary electrophoresis following solid-phase extraction. J. Anal. Methods Chem..

[73] Yun J., Cui C., Zhang S., Zhu J., Peng C., Cai H., Yang X., Hou R. (2021). Use of headspace GC/MS combined with chemometric analysis to identify the geographic origins of black tea. Food Chem..

[74] Wang J., Fu Y., Chen J., Wang F., Feng Z., Yin J., Zeng L., Xu Y. (2022). Effects of baking treatment on the sensory quality and physicochemical properties of green tea with different processing methods. Food Chem..

[75] Ye Y., Yan W., Peng L., Zhou J., He J., Zhang N. (2023). Insights into key quality components in Se-Enriched green tea and their relationship with Selenium. Food Res. Int..

[76] Singh A., Raghuvanshi R.S., Bhatnagar A. (2021). Herbal tea formulation using different flavoured herbs with dried corn silk powder and its sensory and phytochemical analysis. Syst. Microbiol. Biomanuf..

